# On Diseases of Menstruation and Ovarian Inflammation, in Connexion with Sterility, Pelvic Tumors, and Affections of the Womb

**Published:** 1851-08

**Authors:** 


### REVIEWS.

---

ART. IV.—*On Diseases of Menstruation and Ovarian Inflammation, in connexion with Sterility, Pelvic Tumors, and Affections of the Womb.* By EDWARD JOHN TILT, M.D., Physician to the Farrington General Dispensary, and to the Paddington Free Dispensary for the Diseases of Women and Children. "Omne animal ab ovo." New York: Samuel S. & William Wood, 261 Pearl Street. 1851. 12mo. pp. 286.

We have here given the title of a work which proposes to do for the ovaria what the great work of Laennec did for the chest, and which strikes us as one of the most important that has been issued from the press in a long time. "Disordered menstruation"—how vague is the idea which this phrase conveys! Amenorrhea, dysmenorrhea, leucorrhea, hysteria—what is the conception formed by the physician, for the most part, of the pathological condition in which they have their origin? What purely empirical practice has obtained in such affections! Brandy-and-water, camphor, chloroform, hartshorn, valerian, asafetida, or tincture of guiacum, or of cantharides, internal or external stimulants—mere palliatives, or, at most, specifics. And how unsatisfactory such treatment has often proved, the experience of every practitioner painfully testifies.

But must it needs be so? May not the seat and nature of these affections be revealed, and having been ascertained, may not their treatment proceed upon sound scientific principles? Take any one of them and see upon how many morbid states they may depend. Dysmenorrhea, for example, may indicate subacute ovaritis, ovarian peritonitis, effusion of the ovum and menstrual blood into the peritoneum, a neuralgic ovarian affection, an undersized womb, or deviations of that viscus, inflammation of its body, or of the inner surface, producing false membranes, stricture of the neck of the uterus, or its induration, ulceration or cancerous affections of the neck of the womb, besides some other diseased conditions enumerated by Dr. Tilt in the work before us. Now, suppose the practitioner sets about a cure before determining the cause of the affection? He may be successful, but there are many chances that he will not. What will his "emmenagogues" do towards the removal of ulceration or stricture of the neck of the womb? It is plain that we are feeling our way in the dark, until we have assured ourselves of the cause of the disorder.

The aim of the writer of this little volume is to give precise information on these subjects, concerning which it cannot be denied our notions have been far too vague. He is convinced "that there is no reason why the flower of woman's lifetime should remain blighted by intolerable misery, if those organs which stamp the physical character of woman were studied as minutely as the other organs of the body, and if the diseases of each particular portion of the organs of reproduction were investigated with adequate perseverance." He has not attempted to treat of all the organic lesions enumerated as causes of diseased menstruation, but has confined himself to the consideration of the organic diseases by which he believes them to be very frequently produced; namely, *inflmmation of the ovaries and oviducts.* To the fullest extent, he adopts the opinion of Neumann—that "of all the organs of the human frame, none are so often affected by disease as the ovaries," and that suppressed menstruation, a frequent cause of sterility, can generally be traced to disease of those organs.

He holds that amenorrhea, dysmenorrhea, menorrhagia and hysteria are often mere symptoms of subacute ovaritis," which he defines to be—

"Swelling of the ovaria, with increase of heat, and pain upon pressure, accompanied by intermittent or permanent pain or uneasiness in the ovarian region, radiating to the loins and thighs, and producing, acccording to the constitution of the patient, an arrest of menstruation, or its profuse flow, intense local pain, or hysterical symptoms."

The author first treats of the pathological anatomy of ovaritis, and then of its causes; we shall pass at once to the consideration of the latter, which are divided into predisposing and exciting. He says—

"The principal predisposing cause is to be found in the nature and function of the genital organs; for although in woman the ovary is, anatomically speaking, separated from the oviducts, excepting during the first few months of fœtal life, (Meckel and Rosenmuller,) still, in a physiological point of view, the generative intestine is *one* in woman, as it is, anatomically, in many of the lower animals: and whenever these organs are called into functional activity, they unite and become as one organ. Thus, during menstruation, and the orgasm of sexual intercourse, the Fallopian tubes obey an elective impulse, in virtue of which the fimbriated extremities embrace that particular part of the ovaries whence an ovule is to escape, so as to receive it, and the fluids by which it is accompanied,—a fact which has been repeatedly noticed in women dying during menstruation."

Again:

"The periodical congestion of the ovaries is an acknowledged fact, and was strikingly exhibited in a woman affected with hernia of the ovary, which was always observed to become larger immediately before the catamenia, and to diminish on their cessation, (Verdier,

*Traité des Hernies* 1840,) We may therefore admit, that if by any cause this state of congestion were carried to a greater degree than ordinary, or protracted beyond the usual time, general inflammation might attack the organ itself, having its source in the local irritation of which the ovaries are the seat at each menstrual period; and we find accordingly, that in many of the published cases of ovaritis the disease comes on at the time, and instead of the menstrual discharge. Among the predisposing causes, one of the first to be mentioned is, constitution. The disease may indeed occur in all constitutions, but does so more particularly in women who are nervous, irritable, hysterical, and of a scrofulous habit. Girls with long eyelashes, blue sclerotica, and irregular menstruation, have been found most frequently attacked with it, by Burns, Jepherson, Copland, Boivin, and Dugès; but, we have not been able to ascertain the truth of an observation made by Retzius, that women of a certain age, who have borne children and have not suckled, are often attacked with ovaritis. Let us suppose the phenomena of menstruation taking place in one of those delicate girls whose constitution we have indicated—who may perhaps, in her childhood, have been subject to mesenteric deposit, or tubercular peritonitis, not uncommon in children, followed by adhesions of the uterine appendages, and a swollen state of the ovaries; and let us point out what may be the result (in such cases) of the fulfilment of the ovarian function. The first establishment of the menstrual periods cannot take place without the chance of serious disorders, and its return is often attended by the painful symptoms hereafter to be described. Marriage gives an additional impulse to the morbidly disposed ovaries. If, by conception, the ovaries are placed in contact with their final stimulus, this may awaken in them a diseased action, which otherwise might have remained dormant for a time, or have completely disappeared. Abortion is not unfrequently brought on by the nervous ovarian impulse soliciting the expulsion of the fœtus; or the uterus may be bound down by adhesions, which preclude the possibility of its expansion. Should childbirth occur, with its attendant determination of fluids to the pelvic organs, how fatal to ovaries predisposed to disease may be this superabundance of materials and vitality with which they are, for a time, entrusted!

Amongst the functional causes of sub-acute ovaritis, we have alluded to sexual intercourse. Let us consider its excess, or privation, or its intemperate exercise. The excessive use of this stimulus is not unfrequently a cause of sub-acute ovaritis in newly-married women, as the effect of the first impression of a novel stimulus, and its imprudent indulgence. But it is more especially the sequel of the culpable and inordinate exercise of intercourse, as seen in women in every respect unfortunate. Walter and Renaudin state, as the result of their experience, that the ovaries of prostitutes are seldom without some morbid lesions, and Dr. Oldham has lately confirmed their assertion by describing these lesions, which are those of ovaritis. The privation of sexual stimulus is no doubt a cause of certain forms of sub-acute ovaritis; whether we consider its absolute privation in healthy women, whose feelings and passions are strong, or its sudden denial to those accustomed to its indulgence, as in young widows, whom Hildenbrand considers to be often attacked with this complaint, or as in prostitutes when placed in confinement. In such cases the cerebro-spinal sympathies are called into active play, and hysteria masks its local cause. Marriage late in life is sometimes of itself a sufficient cause of sub-acute ovaritis. It seems as if the ovaria, having been debarred their proper stimulus when most needed, become so accustomed to the privation, that when the stimulus is at last presented to them it produces a morbid impression. Sub-acute ovaritis is also one of the pathological elements of that state truly described as the critical time in the life of a woman, and then, in most cases it reacts on the uterus so as to produce those sudden floodings which so often terminate menstruation. If this be not the case, the periodical congestion, which has lasted for so many years, does not at once subside; it still exists long after the menstrual flow has ceased; and as this ovarian congestion is not relieved by its accustomed discharge, the ovaries are liable to inflammation, if such a result be not carefully warded off by repeated purgatives and judicious bleeding, according to the practice, of our medical forefathers--a practice, perhaps, too much neglected in our own day. This crisis in female life is particularly dangerous, both to those involuntary nuns of a society overstocked with women, who have impatiently borne the burden of their virginity, and also to those who have given themselves up to excesses of sexual indulgence. We cannot close the catalogue of predisposing causes without including certain influences, which we shall call moral causes, for want of a better name. They are not tangible, it is true, but they are too important to be overlooked. We allude to all those excitements which tend to exaggerate the impulse of unsatisfied desires—desires, which, though natural in themselves, have been pampered by bodily and mental inactivity, and unduly excited by thoughts, books, pictures, conversation, music, and the fascinations of social intercourse—burning desires, which cannot be quenched by their legitimate satisfaction—at least in our capitals, on account of the greater proportion of marriageable women than that of men."

Dr. Tilt has found the left ovary to be affected by inflammation more frequently than the right, in the proportion of 12 to 5. In ovarian dropsy, on the other hand, it is the right ovary that is generally diseased.

Among the causes of ovarian as well as uterine inflammation in women newly married, Roux has pointed out congenital shortness of the vagina.

The *exciting* causes of ovaritis are numerous, some of which are mechanical, as falls on the feet, knees, or sacrum, jolting on horseback, particularly immediately after menstruation, and violence done during parturition. The instruments sometimes necessarily used in delivery are an admitted cause, and any disproportion between the child's head and the pelvis of the mother, may be productive of the result. It is more likely to follow a first confinement, 25 out of 45 cases of pelvic tumors, reported by Mr. Bell, and 15 out of 32, reported by Mr. Taylor, having occurred in primiparæ.

Among another class of causes may be mentioned styptic injections, employed to stop flooding in the parturient woman, retention or suppression of the catamenia, metritis, the inflammation of the womb being transmitted to the ovary, ulceration of the cervix-uteri, and retroversion of the womb. Retention or suppression of the menses may be either the cause or one of the symptoms of ovaritis. Retention may be—

"First, congenital, as in those numerous cases where it is the result of occlusion by the hymeneal membrane, or of the uterine aperture. Second, it may be accidental, being produced by the blocking up of the passage of the vagina, resulting from parturition, or the pressure of tumors, (as in a case related by Dugès.) It may depend on the gluing together of the os uteri after parturition, or on the imprudent cauterization of its internal surface, and also on the inflammatory tumefaction or the spasmodic contraction of the cervix. The inflammatory tumefaction and spasmodic contraction of the os uteri are most frequently owing to cold applied internally, by taking ices, or a draught of cold water; or externally, by its sudden or prolonged impression on the feet and hands, or on the whole body, by the retaining of wet clothes; and the mode of action of this agent has been shown by the painful colics and prodromi of peritonitis which have sometimes immediately followed the introduction of a cold speculum. Venesection, drastic purgatives, and emetics (when given during menstruation or immediately before) have often been known to produce suppression, and so has sexual intercourse. Any general disturbance of the circulation, such as fevers with or without inflammation, produce the same effect; so may any violent perturbation, mental or moral, occasioned by sudden joy, grief, or anger. When these causes occur on the approach of menstruation, the suspension of the impending flow is followed by sub-acute ovaritis, accompanied by dysmenorrhœa, or hysterical symptoms. When, on the other hand, they operate during the menstrual flow, the sub-acute ovaritis they may produce is attended by engorgement of the uterus, which is accounted for by the active congestion of its tissues, and the retention of blood in its irritated cavity."

Retention or suppression of the menses has a twofold influence in the production of ovaritis;—"first, the retention of what was to have been excreted, and the consequent congestion of the organs which secrete the menstrual discharge; secondly, by the arrest of the ovarian discharge, and the subsequent oppression of the system by some reflected influence of a nervous kind."

The author continues—

"1. The mechanical effects of retention of the menstrual flow are, the repletion of the womb and the Fallopian tubes: the distension by, and necessary reaction of, the ovary and Fallopian tubes against a menstrual flow too abundant in quantity, and some times rendered noxious by its prolonged sojourn in the human body. It has even been proved that in some rare cases the distention of the Fallopian tubes is so great as to detach the fimbriated extremity from the ovarium, allowing a flow of blood into the peritonæum, and thus producing peritonitis.--*Archives Gen. de Med.* 1848.

2. The suppression of the menstrual flow also acts by the arrest of the ovarian nervous discharge which it involves, and the consequent oppression of the system by some reflex nervous influence. For how can we suppose that sudden death, in the midst of the most alarming symptoms of convulsions and delirium, could be solely produced by the retention of a few ounces of blood; or, if we could even admit such an explanation, what should we do under circumstances similar to those of authentic cases wherein the same symptoms have been brought on by the suspension of the *impending* menstrual flow?"

There can be no doubt, that inflammation of the uterus is a frequent cause of ovaritis, just as inflammation of the duodenum gives rise to hepatitis, or that of the urethra to inflammation of the testicle. Thus, in the case of a young lady mentioned by Dr. Tilt, there was enlargement of the ovary to triple its size, associated with ulceration of the cervix-uteri; and on the cure of the latter by cauterization with the red-hot iron, the tumor disappeared. In another case, in which the two conditions existed together, Lisfranc amputated the neck of the womb, and six years afterwards the tumor had not increased. Such, indeed, is the influence of the cervix-uteri over the ovaria, that Dr. H. Bennet ascribes to ulcerations of this part the power of inducing every variety of diseased menstruation.

The next chapter treats of the symptoms of subacute ovaritis.

"The patient experiences a dull pain in the ovarian region, often imperceptible when she is in a state of repose, but brought on by walking, riding, by any sudden movement, or even by pressure on the side. The pain is also increased by the act of straightening the thigh upon the pelvis, as in the erect posture, by which the integuments are put upon the stretch, and pressure is thus exerted over the part. Some patients are unable to maintain the erect posture without resting the foot of the side affected on a stool, so as to keep the thigh more or less bent upon the pelvis, whereby the integuments, &c. are relaxed. Radiating from the ovarian region, the pains are felt across the loins; they descend towards the thighs and fundament, and are of a dull, dragging, heavy, and sometimes of an overwhelming nature. They are distinguished by the patient from other pains resembling colic, and which depend on uterine contractions, although both species of pain may be experienced at the same time; they are likewise to be distinguished from those *superficial* pains which are caused by reflex nervous action, and which so frequently accompany every species of disorder of the organs of generation. They are, however, seldom so acute as to induce the patient to seek for advice. She may submit to them for years, but should she find them so wearisome to mind and body as to be led to seek advice upon her case, she is frequently treated for uterine disease. This is owing to the opinion, adopted by Hippocrates, and still too implicitly believed, that the uterus is the principal organ of the generative system, and that to the morbid condition of this organ are to be attributed almost all the sufferings of a woman. Should the patient be married, connexion awakens and renders more or less acute the pains we have described. Ocular inspection, and an attentive manual examination, however, will, in some instances, prove that it is not painful when touched, nor does it present much appearance of disease. In sub-acute ovaritis, the hands placed on the iliac regions can sometimes detect an increase of heat; but these symptoms of ovarian inflammation are overlooked, or attributed to disease of the womb, inflammation of its neck, or to that scape-goat of uterine pathology, only known in England, and called irritable uterus—a disease regarded as neuralgia by some, as a form of dysmenorrhœa by others, and which, having the same symptoms as sub-acute ovaritis, we suppose sometimes to be one of the legionic names of that disease. The late Dr. Ingleby noticed that the descent of the ovaries on the vagina produced in one of his patients all the symptoms of the disease called irritable uterus."

After enumerating the *common symptoms* of subacute ovaritis, the author goes on to consider the affection under several types, as the amenorrheal, dysmenorrheal, menorrhagic, and hysterical. We quote what is said under the second head:—

"The frequent dependence of painful menstruation on sub-acute ovaritis has been generally recognized, and is now admitted by Drs. Oldham, Rigby, Ashwell, Coley, and others too numerous to recount.

In addition to the symptoms before described, the intensity of the pain becomes most distressing, and it frequently commences several days before the impeded menstrual flow, showing that the pain does not depend on its arrest, but on the effect of ovulation supervening upon a morbid process going on in the ovaries. This assertion is confirmed by Dr. Ashwell, who says, 'Dull and heavy pains in the region of the ovaries, lasting for months, are the consequence of their chronic (sub-acute) inflammation. I mention the circumstance, because they are too often regarded as neuralgic, and treated accordingly, painful menstruation and sterility being their results. If any constitution is more liable than another to this termination, it is also the lymphatic, or that which coincides with a marked predisposition to scrofula.'

The action of sub-acute ovaritis in the production of dysmenorrhœa is twofold.

1. Sub-acute ovaritis may of itself produce dysmenorrhœa, as a simple result of the process of mordid ovulation, and not by the agency of any appreciable inflammation of the womb, or of its neck, and without any appearance of false membrane in the catamenia. This is what we have seen, and believe to be frequent.

2. Ovaritis, as Dr. Oldham has well shown, often causes dysmenorrhœa by determining hypertrophy of the uterus, inflammation of its neck, and a diptheritic exudation from its mucous surface. We know that the ovaries, in virtue of their governing influence over the uterus, induce periodically a state of vascular turgescence in the walls of this organ; and it is not surprizing to find that ovaritis frequently induces the exaggeration of this physiological state, or the inflammation of the inner surface of the womb and of its neck; thereby transforming the thin, transparent mucous membrane of the womb in a thick, soft cribriform membrane, and producing the retention or painful excretion of the catamenia, which are mingled with pseudo-decidual membranes.

We cannot refrain from giving a passage from Dr. Oldham's interesting observations:—

"The uterine decidua is formed under the influence of an action going on in the ovary, so the membranous dysmenorrhœa is not primarily an affection of the womb, but of the ovary. In healthy menstruation the congestion of the ovary the engorgement of the womb, the opening of the veins on the surface of the cavity of the womb, and the flux of blood, are all in harmony, the latter being, so to speak, the resolution of the former. But when the ovaries are unduly excited, as, for instance, from the prevalence of one or more of the numerous ways in which sexual feelings may influence them, then the uterine glands sympathetically enlarge, the lining membrane of the womb becomes raised and the body of the womb swells out. This change in the mucous membrane goes on during the interval between the monthly periods, and when the flow begins, the new formation is cast off, and the uterus, in the act of detaching and expelling it, becomes the seat of very painful contractions."

The actual presence of ovaritis is to be ascertained by an abdominal examination, and exploration per vaginam and per rectum, the success of which will depend upon the skill with which the practitioner conducts them. A great obstacle in the way of the latter is the insuperable repugnance of most females in our country to such manipulations, which, in fact, is likely to limit the investigation, as a general rule, to the first procedure.

In the fourth chapter, the author discusses the terminations of subacute ovaritis. The most interesting of these is *sterility*, which the inflammation may produce, "1, by accelerating the shedding of imperfectly developed ova; 2, by the retention of blighted ova; 3, by impeding their transmission from the ovaries to the uterus." The following observations on this point are of so much practical importance that we copy them at length:

1. We are sometimes consulted by delicate females lately married, who present all the *common* symptoms of sub-acute ovaritis, but in whom menstruation returns every three weeks, or even every fortnight; cases of what may be called *remittent* menstruation, wherein the recovery from one menstrual epoch is almost immediately followed by the recommencement of the same process. In such cases, the excitation of ovulation exceeds the limits of the physiological type, and the ovule is shed before it has acquired its full maturity. In other words, the ovule perishes by *ovarian* abortion in the beginning of its intended career, in the same way that it might perish, at a later period of its existence, by *uterine* abortion—an extension of the word abortion warranted by the similarity of the phenomena in both cases. We have thus ascribed a due importance and a precise signification to those lesions of the parietes of the vesicles which merely expedite the exit of the immature ovule from its ovarian utricle. But we are the first to admit that our explanation of the facts alluded to rest entirely on the truth of a relation of cause and effect between ovulation and menstruation—a relation which, however probable, has not yet been completely traced.

2. The blighting of the ova while they are still contained in the Graaffian follicle cannot be denied. That De Graaff has not failed to point out this possible fate of the human germ, its disease, adhesion, and absorption, in the midst of the inflammatory action, will be readily admitted, and a positive proof may be seen. (*Med. Chirur. Trans.*, t. vi.) Dr. Edward Stanlen, on opening the body of a woman who committed suicide, found the left ovary voluminous, and on one of its sides a small elevation; in this elevation, but *under the ovarian peritonæum*, was a minute cyst, containing an ovum about the size of a cherry-stone—it adhered to the cyst in two-thirds of its circumference, and was distinctly formed of the chorion and amnion, but offered no trace of a fœtus. A more common cause of sterility is to be found in the frequent inflammation of that portion of the peritonæum which covers the organs of procreation. This frequency has not been overlooked by Dr. Robert Lee. The adhesions, he observes, between the ovarian and the Fallopian tubes, which are so frequently met with in examining the bodies of women of different ages and conditions, prove that slight attacks of inflammation of the peritonæal coat of the ovaria are not of rare occurrence, and that their presence is seldom discovered during life. Dr. Carswell also bears witness to the frequency of these incontestable proofs of inflammation in the same region :—

"The adhesion which form between the uterus, Fallopian tubes, and ovaries, and the surrounding parts, are much more productive of serious effects than in any other region of the body; and in order to give additional importance to the study of them, I may observe that they are a not unfrequent, and certainly one of the most obvious causes of sterility. They produce, according to their situation and mode of attachment, either anteversion or retroversion of the uterus; they fix the Fallopian tubes in situations in which the fimbriated extremities cannot reach the ovaris, or they envelop the fimbriated extremities in such a manner as to render them quite impervious, (which is always the cause of dropsy of these tubes,) or lastly, they cover the ovaries so completely that impregnation is rendered impossible."

We therefore conclude that, in many cases, dysmenorrhœa is another name for ovarian peritonitis, and that sterility is often the result of the thickening of the peritonæum, of its being clothed with false membranes, and also of the destruction of the fimbria. And we believe with our friend Dr. Mercier, (who has lately published an interesting memoir on the subject,) that this inflammation occurs oftener than is generally supposed, and is transmitted by continuity from the womb by the Fallopian tubes; thereby explaining the frequency of sterility in prostitutes, amongst whom Parent--Duchâtelet says, scarcely six accouchments per thousand take place in the course of the year, (tom. i. p. 230.) We must also observe, that many women are sterile from this cause, although they are not considered to be so because they have previously borne children. Professor Richerand has remarked, that generally young women who complain of sterility have suffered from previous attacks of "inflammation of the bowels."

3. It is generally admitted that, without being permanently obliterated, the Fallopian tubes may be blocked up by mucus; and, lately, Drs. Fleetwood, Churchill, Copland, Hamilton, and others, have admitted the fact. And it is evident that such an obstruction must impede as well the descent of the ovum, as it does the ascent of the seminal fluid. Whether or not this condition furnishes any direct therapeutical consideration we shall leave for future investigation, and proceed to inquire whether subacute ovaritis produces disease of the womb, and by what means."

Another termination is inflammation of the uterus, the reality of which has been established by the observations of several able authors.

In the next chapter, the *treatment* is given, and as that which Dr. Tilt has found successful is well exemplified by his first case cited under this head, we present it in detail:—

"*Sub-acute ovaritis producing sterility; cure followed by pregnancy.* When practising in Paris, in 1844, we were consulted by a gentleman, about thirty years of age, presenting every appearance of good health, who told us that his wife was in her twentyfourth year, that at the age of fifteen she menstruated for the first time, but that this function had always been accompanied by pain, and was frequently irregular in the time of its appearance. He had been married five years, and since then the menstrual discharge had been more regular, but accompanied by a great increase of pain. She was seldom subject to leucorrhœa, but sexual indulgence was sometimes painful. For the last year, various means of medical relief had been tried; but with so little success, that her husbaud said he was not induced to consult us for his wife in the hope of our being able to relieve her monthly suffering, but to inquire if there were any remedy for sterility. The lady presented all the appearance of a lymphatic constitution; she looked delicate but was apparently in tolerable health; she did not expect to be unwell for the next fortnight, and she was not then in pain; but on rapidly depressing the ovarion regions with the united tips of the fingers, we produced a pain similar to that she experienced when menstruating. On examining by vagina, we received an indistinct perception of a small tumor, which we took for the right ovary; but on making a rectal examination, we distinctly felt both ovaries, each being swolen to about two inches in the long diameter. They were painful on pressure. Having ascertained the tumefied state of the ovaria, and their tenderness on pressure, and bearing in mind the previous history of the patient, we considered them sub-acutely inflamed. We determined, however, to do nothing previously to the next monthly period, so that we might judge of the nature of her sufferings, and afterwards have full three weeks to alleviate them. A few days after, she was suffering from all the symptoms of dysmenorrhœa; the pain, on pressing the ovarian regions, was greater; and, on examining through the rectum, the ovaria were found still larger and more painful. When the period was over, we began the treatment, by applying eight leeches over each ovarian region; the leech-bites being healed, we applied over the same region a blister, five inches in length; the cuticle was not removed, and three days after, when the skin was healed, we ordered the same region to be carefully rubbed for ten minutes, morning and night, with a portion, about the size of a walnut, of the following ointment: ung. hydrag. ℥j, ext. belladonnæ ʒj, ext. hyoscyami ʒj, camph. (solut. in spirit.) gr. x; the abdomen to be afterwards covered with flannel, without removing the ointment. We also prescribed enemata of aquæ camp, ℥ xv, aquæ lauri cerasi, ʒvi; sometimes adding tinct. hyoscyami, ʒiii. A third of this quantity was injected into the rectum three times a-day, the chill having been first taken off, so that it might be as much as possible, if not entirely retained. Due attention was paid to the regularity of the bowels; mercury being avoided, and saline purgatives preferred. For the first few days, until the blistered surfaces were healed, the patient left her bed only to recline on the sofa; afterward she was allowed to take exercise as usual, and her strength was kept up by a genorous diet. Abstinence from the nuptial bed was strictly enjoined. On examining by the rectum a few days before the expected time, we found the ovaria diminished in size, but still painful to the touch. The next menstrual period was accompanied by the usual dysmenorrhœal symptoms; but the patient said that she had suffered less than she had ever done since her marriage. When menstruation had ceased, we subjected her to exactly the same treatment, and her sufferings were again diminished during the ensuing menstruation. She submitted to the same course a third time; and on exploration, we found that the ovaries had resumed their usual size, and that pressure was not accompanied by pain. The third menstruation since the beginning of the treatment was attended by little pain. We discontinued the leeches, blister, and ointment, but advised the regular continuation of the enemata. We permitted cohabitation, at the same time recommending moderation to her husband. Four months after this, our patient was pregnant, and in due time was delivered of a fine boy."

This case comprises all that it is necessary to say on the subject of treatment, except that the affection is often most obstinate, and must be attacked for months after each menstrual period by some such remedies as are above pointed out.

We have not room to give any account of the other part of this work, which treats of acute ovaritis, but what has been shown is sufficient, we think, to direct the attention of our readers seriously to the subject. It may be that the author over-estimates the frequency and influence of these affections, but that we have needed light in reference to them every practitioner will admit. Dr. Tilt is unquestionably in the right path, and the prosecution of his researches must lead to valuable results. Our conviction is that he has already arrived at useful conclusions, and that his work is an important contribution to our knowledge concerning a most interesting class of maladies. Y.

